# Prevalence and predictors of vision impairment among older adults in India: evidence from LASI, 2017–18

**DOI:** 10.1186/s12886-023-03009-w

**Published:** 2023-06-05

**Authors:** Shobhit Srivastava, Manish Kumar, T. Muhammad, Paramita Debnath

**Affiliations:** International Institute for Population Sciences, Mumbai, Maharashtra 400088 India

**Keywords:** Vision impairment, Chronic condition, Socioeconomic, Older adult, India

## Abstract

**Background:**

Older adults experience a natural decline in health, physical and cognitive functionality, and vision impairment (VI) is one among them and has become an increasing health concern worldwide. The present study assessed the association of chronic morbidities such as diabetes, hypertension, stroke, heart diseases and various socioeconomic factors with VI among older Indian adults.

**Methods:**

Data for this study were derived from the nationally-representative Longitudinal Ageing Study in India (LASI), wave-1 (2017–18). VI was assessed using the cut-off of visual acuity worse than 20/80, and additional analysis was carried out using the definition of VI with a cut-off of visual acuity worse than 20/63. Descriptive statistics along with cross-tabulation were presented in the study. Proportion test was used to evaluate the significance level for sex differentials in VI among older adults. Additionally, multivariable logistic regression analysis was conducted to explore the factors associated with VI among older adults.

**Results:**

About 33.8% of males and 40% of females suffered from VI in India (visual acuity worse than 20/80). Meghalaya (59.5%) had the highest prevalence for VI among older males followed by Arunachal Pradesh (58.4%) and Tripura (45.2%). Additionally, Arunachal Pradesh (77.4%) had the highest prevalence for VI among females followed by Meghalaya (68.8%) and Delhi (56.1%). Among the health factors, stroke [AOR: 1.20; CI: 1.03–1.53] and hypertension [AOR: 1.12; CI: 1.01–1.22] were the significant risk factors for VI among older adults. Additionally, being oldest old [AOR: 1.58; CI: 1.32–1.89] and divorced/separated/deserted/others [AOR: 1.42; CI: 1.08–1.87] were significantly associated with VI. Moreover, older adults with higher educational status [AOR: 0.42; CI: 0.34, 0.52], currently working [AOR: 0.77; CI: 0.67, 0.88], from urban areas [AOR: 0.86; CI: 0.76–0.98] and from western region [AOR: 0.55; CI: 0.48–0.64] had lower odds of VI in this study.

**Conclusion:**

This study identified higher rates of VI among those who are diagnosed with hypertension or stroke, currently unmarried, socioeconomically poorer, less educated and urban resident older people that can inform strategies to engage high risk groups. The findings also suggest that specific interventions that promote active aging are required for those who are socioeconomically disadvantaged as well as visually impaired.

**Supplementary Information:**

The online version contains supplementary material available at 10.1186/s12886-023-03009-w.

## Background

Older adults experience a natural decline in health, physical and cognitive functionality [1]. Visual impairment (VI) is one among them and has become an increasing health concern worldwide [2]. Globally, about 295 million people of all ages have problems related to vision and 43 million people are estimated to be blind, although constant global initiatives had extended reduction in cases of avoidable blindness, but the prevalence of low vision is expected to double during the next 30 years and the global burden is expected to increase in line as the population ages [3, 4]. The top three causes of most severe vision loss among adults aged 50 years and above are cataract, refractive errors and glaucoma that are avoidable through comprehensive eye tests, surgery, medications and spectacle corrections early in the disease progression which can delay or prevent 80% of the cases of VI [5]. Studies suggested the prevalence of blindness due to mentioned causes to be substantially higher in countries like East Asia, South East Asia and Sub-Saharan Africa compared to high-income sub-regions (2). Low vision on the other hand, has significant impact on lives of millions of older adults and poses serious challenges on person’s independence, mobility, usual activities of daily living and poor quality of life [6, 7]. It is therefore a pressing issue that needs urgent attention because of its complex and far-reaching impacts on both older adults and society as a whole especially in developing countries [8].

The effect of deteriorating general health with old age is often interwoven with loss/weakening of vision [9]. Multiple comorbidities existing with VI generally appear to have important implication on health care and rehabilitation services for older adults [10]. Notably, diabetes, hypertension, heart diseases are few most prevalent conditions among older adults [11]. Some of these comorbidities are also frequently observed in older adults with VI [10]. Studies have also suggested other associated factors such as poverty, inadequate access to basic amenities and malnutrition to be significant for low vision/complete loss of vision. Similarly, several cross-sectional studies in India, indicated prevalence of VI and higher risk of morbidities and mortality, widely distinctive based on different sex and socio-economic circumstances of an older adult [12], for instance, older women are often more likely to have VI [13], while, evidences are inconclusive as some suggest women are over represented in the VI group compared to men [10]. There is substantial evidence from developing countries, where, only small proportion of older adults aged 65 and above have financial independence, higher education and employment as factors consistently associated with being at higher risk of VI [8].

So far, VI had been mostly characterised as an age-related problem due to systemic and sensory changes majorly influencing loss of vision among older people [14]. The information on various risk factors of VI among older adults is important to quantify the unmet needs for eye care services, especially in resource-limited settings including India, so that early detection, diagnosis and treatment can be facilitated for older individuals with reduced vision in a community. The current study assessed the association of chronic morbidities such as diabetes, hypertension, stroke, heart diseases and various socioeconomic factors with VI using data from the Longitudinal Ageing Study in India (LASI, wave-1). Also, regional differences in the prevalence of VI among older adults are explored in this study.

## Methods

### Data

Data for this study were derived from the nationally-representative LASI, wave-1 [15]. LASI is a full-scale national survey of scientific investigation of the health, economic, and social determinants and consequences of population aging in India, conducted in 2017–18. The LASI is a nationally representative survey over 72,000 older adults age 45 and above across all states and union territories of India. The main objective of the survey is to study the health status and the social and economic well-being of older adults in India. LASI adopted a multistage stratified area probability cluster sampling design to arrive at the eventual units of observation: older adults age 45 and above and their spouses irrespective of age [15]. The survey adopted a three-stage sampling design in rural areas and a four-stage sampling design in urban areas. In each state/UT, the first stage involved the selection of Primary Sampling Units (PSUs), that is, sub-districts (Tehsils/Talukas), and the second stage involved the selection of villages in rural areas and wards in urban areas in the selected PSUs. In rural areas, households were selected from selected villages in the third stage. However, sampling in urban areas involved an additional stage [15]. Specifically, in the third stage, one Census Enumeration Block (CEB) was randomly selected in each in urban area. In the fourth stage, households were selected from this CEB. The detailed methodology, with the complete information on the survey design and data collection, was published in the survey report [15]. The present study is conducted on the eligible respondent’s age 60 years and above. The total sample size for the present study is 31,464 (15,098 male and 16,366 female) elders aged 60 years and above [15].

### Variable description

#### Outcome variable

In LASI, for all participants, near vision and distance vision was measured for both eyes with the best possible correction available. According to the world health organization (WHO), International Classification of Diseases (ICD)- 10th Revision: vision impairment is defined as presenting visual acuity of less than 6/18 in the better eye with available correction. A screen of computer-assisted personal interviewing (mini laptop CAPI device)—based tumbling E log MAR (Logarithm of the Minimum Angle of Resolution) chart was used for the vision-related measurements [15]. As per the standard protocol, the near vision and distance vision was measured at 40 cm and 3 m, respectively [16]. In the E log MAR chart, the scale orientations used for near vision were 20/20, 20/25, 20/32, 20/40, 20/50, 20/63, 20/80, 20/125, 20/160, 20/250, 20/320, and 20/400, and for distance vision were 20/20, 20/25, 20/32, 20/40, 20/50, 20/63, 20/80, 20/125, 20/160, 20/250, and 20/320. The low near vision is defined as the “near vision equal to or poorer than 20/80 and equal to or better than 20/400 in the better eye”. On the other hand, low distant vision is defined as the “distance vision equal to or poorer than 20/80 and equal to or better than 20/200 in the better eye” [15, 17]. Finally, visual impairment (VI) includes both the near and distance low vision, and older adults were categorised as visually impaired if they had either low near vision or low distant vision. For the sensitivity analysis, VI was also measured using the cut-point of a visual acuity worse than 20/63, according to the World Health Organization criteria (Supplementary material). Moreover, a visual acuity of less than 20/400 (3/60) in the better eye and being unable to count fingers or perceive light have been considered blind.

### Covariates

Diabetes, hypertension, stroke and heart disease were coded as no and yes. The variables were created based on the question “Has any health professional ever diagnosed you with the following diseases?” The response was categorized as 0 “no” and 1 “yes”. Such patient-reported outcome measures (PROM) do have their salience in this particular context [18].

According to the studies mentioned above, several socio-demographic variables were selected and included in this study. Age was categorized as young old (60–69 years), old-old (70–79 years) and oldest-old (80 + years). Sex was categorized as male and female. Education was categorized as no education, primary, secondary and higher. Marital status was categorized as currently married, widowed and others (divorced/ separated/ deserted/ never married). Working status was categorized as working, retired and not working [19]. Current tobacco (smoke and chew) users were categorized as no and yes. The ever use of alcohol in lifetime was categorised as no and yes.

Monthly per capita expenditure (MPCE) quintile was estimated using household consumption data. The details of the measure are provided in the survey report [15]. The variable was divided into five quintiles i.e., from poorest to richest. Religion was categorized as Hindu, Muslim, Christian and Others. Caste was categorized as Scheduled Tribe, Scheduled Caste, Other Backward Class and others. The Scheduled Caste includes a group of population which is socially segregated and financially/economically by their low status as per Hindu caste hierarchy. The Scheduled Castes (SCs) and Scheduled Tribes (STs) are among the most disadvantaged socio-economic groups in India. The OBC are the group of people who were identified as “educationally, economically and socially backward”. The OBCs are considered low in traditional caste hierarchy [20]. The “others” category is identified as people having higher social status. Place of residence was categorized as rural and urban. Regions were coded as North, Central, East, Northeast, West and South.

### Statistical analysis

Descriptive statistics along with cross-tabulation were presented in the present study. Proportion test was used to evaluate the significance level for gender differentials in VI among older adults [21]. Additionally, multivariable logistic regression analysis [22] was used to establish the association between the outcome variable (VI) and other explanatory variables. The estimates provided are adjusted odds ratio (AOR) as they will be adjusted for the selected background characteristics.

The binary logistic regression model is usually put into a more compact form as follows:

$$\mathrm{Logit }\left[\mathrm{P}\left(\mathrm{Y}=1\right)\right]={\beta }_{0}+\beta *X$$

The parameter ${\beta }_{0}$ estimates the log odds of VI for the reference group, while $\beta$ estimates the maximum likelihood, the differential log odds of VI associated with a set of predictors X, as compared to the reference group. Variance inflation factor (VIF) was generated in STATA 14 [23] to check the multicollinearity and there was no evidence of multicollinearity in the variables used [24, 25] (Supplementary Table S1). Additionally, individual weights were used which were present in the dataset to make the estimates nationally representative.

## Results

Table 1 represents sample characteristics of the study population. Nearly 16.3% of older males and 14.8% of older females suffered from diabetes. About 30.8% of older males and 38.9% of older females suffered from hypertension. Nearly 3.3% and 5.9% of older males and 2.1% and 4.2% of older females suffered from stroke and heart diseases respectively. Almost, 36.3% of older males and 69% of older female were not educated, whereas, about 12.1 older males and 3.5% older females had higher educational status. Nearly, 15.2% of older males and 51.5% of older females were widowed. Almost 5% and 49% of older males and older females never worked. About 24.5% and 23.3% of older males and 4.3% and 15.5% of older females smoked tobacco and chewed tobacco, respectively. Also, nearly 31.3% of older males and 4.2% of older females consumed alcohol.

**Table 1:** Sample characteristics of the study population, India, LASI Wave 1, 2017–18

Background characteristics	Male		Female		Total	
	**n**	**%**	**n**	**%**	**n**	**%**
**Diabetes**						
No	12,594	83.7	13,927	85.2	26,521	84.5
Yes	2,444	16.3	2,416	14.8	4,860	15.5
**Hypertension**						
No	10,401	69.2	9,986	61.1	20,387	65.0
Yes	4,640	30.8	6,355	38.9	10,995	35.0
**Stroke**						
No	14,546	96.7	15,994	97.9	30,540	97.3
Yes	495	3.3	347	2.1	842	2.7
**Heart Disease**						
No	14,155	94.1	15,656	95.8	29,811	95.0
Yes	886	5.9	686	4.2	1,572	5.0
**Age group (in years)**						
Young-old (60–69)	8,961	59.4	10,013	61.2	18,974	60.3
Old-old (70–79)	4,545	30.1	4,556	27.8	9,101	28.9
Oldest-old (80 +)	1,592	10.5	1,797	11.0	3,389	10.8
**Education**						
No education	5479	36.3	11,410	69.7	16,889	53.7
Primary	4479	29.7	3,081	18.8	7,560	24.0
Secondary	3307	21.9	1,307	8.0	4,614	14.7
Higher	1833	12.1	568	3.5	2,401	7.6
**Marital status**						
Currently married	12,398	82.1	7,522	46.0	19,920	63.3
Widowed	2,293	15.2	8,426	51.5	10,719	34.1
Others^a^	407	2.7	418	2.6	825	2.6
**Working status**						
Never worked	755	5.0	8,021	49.0	8776	27.9
Currently working	6,331	41.9	2,976	18.2	9307	29.6
Not currently working	8,008	53.1	5,365	32.8	13,373	42.5
**Currently smoke tobacco**						
No	11,274	75.5	15,557	95.7	26,831	86.0
Yes	3,667	24.5	706	4.3	4,373	14.0
**Currently chew tobacco**						
No	11,461	76.7	13,749	84.5	25,210	80.8
Yes	3,480	23.3	2,514	15.5	5,994	19.2
**Alcohol consumption**						
No	10,263	68.7	15,583	95.8	25,846	82.8
Yes	4,679	31.3	685	4.2	5,364	17.2
**MPCE quintile**						
Poorest	3,035	20.1	3,449	21.1	6,484	20.6
Poorer	3,068	20.3	3,409	20.8	6,477	20.6
Middle	3,064	20.3	3,352	20.5	6,416	20.4
Richer	2,990	19.8	3,180	19.4	6,170	19.6
Richest	2,941	19.5	2,976	18.2	5,917	18.8
**Religion**						
Hindu	11,078	73.4	11,959	73.1	23,037	73.2
Muslim	1,804	11.9	1,927	11.8	3,731	11.9
Christian	1,468	9.7	1,682	10.3	3,150	10.0
Others^b^	748	5.0	797	4.9	1,545	4.9
**Caste**						
Scheduled Caste	2,448	16.7	2,692	17.1	5,140	16.9
Scheduled Tribe	2,436	16.6	2,737	17.3	5,173	17.0
Other Backward Class	5,781	39.5	6,105	38.7	11,886	39.1
Others	3,970	27.1	4,248	26.9	8,218	27.0
**Place of residence**						
Rural	10,077	66.7	10,648	65.1	20,725	65.9
Urban	5,021	33.3	5,718	34.9	10,739	34.1
**Region**						
North	2,799	18.5	3,013	18.4	5,812	18.5
Central	2,155	14.3	2,107	12.9	4,262	13.6
East	2,863	19.0	2,894	17.7	5,757	18.3
Northeast	1,782	11.8	1,970	12.0	3,752	11.9
West	1,953	12.9	2,350	14.4	4,303	13.7
South	3,546	23.5	4,032	24.6	7,578	24.1
**Overall**	**15,098**	**100.0**	**16,366**	**100.0**	**31,464**	**100.0**

^a^ Includes Divorced/Separated/Deserted/Others

^b^ Includes Sikh, Buddhist/neo-Buddhist, Jain, Jewish, and Parsi/Zoroastrian

Table 2 represents the age-sex adjusted and unadjusted prevalence of VI among older adults in India, 2017–2018 (visual acuity worse than 20/80). About 37.1% of older adult had VI when unadjusted for age and sex. However, about 37.6% of older adult had VI when adjusted for age and sex. As documented in Supplementary Table S1, the values were much higher when VI was measured with a cut-off of worse than 20/63 (64.7% in the unadjusted and 64.1% in the adjusted estimates).

**Table 2:** Age-sex adjusted and unadjusted prevalence of VI among older adults in India, 2017–2018

	**Unadjusted**		**Adjusted**	
	**%**	**CI**	**%**	**CI**
**Overall**	37.1	(36.50, 37.62)	37.6	(37.05, 38.16)
**Diabetes**				
No	38.8	(37.62, 38.85)	38.6	(37.96, 39.18)
Yes	32.1	(28.72, 31.43)	32.4	(30.99, 33.77)
**Hypertension**				
No	38.2	(36.49, 37.88)	38.5	(37.81, 39.20)
Yes	36.8	(35.88, 37.78)	35.9	(34.99, 36.87)
**Stroke**				
No	37.7	(36.41, 37.54)	37.5	(36.98, 38.11)
Yes	40.3	(36.85, 44.15)	40.4	(36.61, 44.13)
**Heart Disease**				
No	38.0	(36.76, 37.91)	37.9	(37.27, 38.42)
Yes	32.9	(29.71, 34.55)	33.5	(31.08, 35.99)
**Education**				
No education	44.3	(41.75, 43.32)	43.3	(42.43, 44.10)
Primary	35.4	(34.39, 36.64)	36.0	(34.84, 37.16)
Secondary	26.4	(24.04, 26.68)	27.6	(25.94, 29.30)
Higher	20.2	(18.68, 22.12)	21.8	(19.45, 24.15)
**Marital status**				
Currently married	34.7	(33.80, 35.19)	36.8	(36.00, 37.60)
Widowed	43.4	(40.10, 42.07)	40.1	(38.86, 41.40)
Others^a^	38.7	(40.36, 47.60)	38.7	(35.12, 42.28)
**Working status**				
Never worked	42.7	(40.49, 42.67)	42.4	(40.45, 44.43)
Currently working	34.0	(31.54, 33.53)	38.3	(36.91, 39.62)
Not currently working	37.1	(36.76, 38.49)	36.7	(35.78, 37.57)
**Currently smoke tobacco**				
No	37.9	(36.91, 38.13)	37.2	(36.58, 37.79)
Yes	36.7	(32.86, 35.79)	40.4	(38.25, 42.44)
**Currently chew tobacco**				
No	37.4	(36.38, 37.64)	37.0	(36.41, 37.65)
Yes	39.1	(36.02, 38.56)	40.2	(38.87, 41.48)
**Alcohol consumption**				
No	37.9	(36.68, 37.92)	36.9	(36.32, 37.56)
Yes	36.8	(34.35, 37.02)	41.7	(39.55, 43.78)
**MPCE quintile**				
Poorest	41.1	(38.99, 41.51)	40.9	(39.68, 42.2)
Poorer	39.8	(38.62, 41.13)	39.6	(38.34, 40.82)
Middle	38.4	(35.33, 37.79)	38.3	(37.04, 39.50)
Richer	36.0	(32.66, 35.14)	35.8	(34.53, 37.02)
Richest	32.9	(32.25, 34.79)	33.0	(31.79, 34.28)
**Religion**				
Hindu	37.2	(36.34, 37.65)	37.1	(36.44, 37.74)
Muslim	35.0	(33.53, 36.75)	35.1	(33.55, 36.74)
Christian	42.7	(40.03, 43.66)	42.2	(40.35, 43.94)
Others^b^	42.3	(38.02, 43.20)	42.0	(39.38, 44.56)
**Caste**				
Scheduled Caste	42.0	(40.23, 43.06)	42.1	(40.73, 43.56)
Scheduled Tribe	43.5	(39.12, 41.93)	43.4	(41.99, 44.79)
Other Backward Class	36.3	(35.36, 37.17)	36.2	(35.32, 37.11)
Others	33.2	(32.57, 34.73)	32.8	(31.77, 33.89)
**Place of residence**				
Rural	40.7	(38.80, 40.19)	40.6	(39.88, 41.26)
Urban	31.8	(30.02, 31.88)	31.7	(30.81, 32.67)
**Region**				
North	39.5	(40.45, 43.1)	39.3	(38.01, 40.60)
Central	35.9	(35.26, 38.31)	35.9	(34.41, 37.43)
East	39.2	(39.10, 41.74)	39.2	(37.93, 40.53)
Northeast	48.2	(47.68, 51.06)	47.6	(45.93, 49.25)
West	29.5	(25.70, 28.54)	29.7	(28.23, 31.14)
South	35.6	(35.52, 37.81)	35.4	(34.25, 36.51)

^a^ Includes Divorced/Separated/Deserted/Others

^b^ Includes Sikh, Buddhist/neo-Buddhist, Jain, Jewish, and Parsi/Zoroastrian

Prevalence (%) of VI among older adults, stratified by sex, according to their background characteristics is presented in Table 3 (visual acuity worse than 20/80). Older males (39.7%) and females (41.5%) who suffered from stroke had higher prevalence of VI. Oldest old males (47.8%) and females (47.4%) had higher prevalence of VI. Older males and females with no formal education had higher prevalence of VI. Older males (44.7%) and females (41.4%) who never worked had higher prevalence of VI. Older males (34.8%) and females (44.5%) who consumed alcohol had higher prevalence of VI. Older male and females from lower socio-economic groups i.e., from poorest MPCE quintile, Scheduled Caste and with a rural residence had higher prevalence of VI. Additionally, older females had significantly higher prevalence of VI than older males (Difference: -6.2%; *p* < 0.001). The prevalence of VI stratified by sex according to the cut-off of visual acuity worse than 20/63 is presented in Table S2.

**Table 3:** Prevalence (%) of VI among older adults according to background characteristics by sex, India, LASI Wave 1, 2017–18

Background characteristics	Male	Female	Difference	*P*-value
	**%**	**%**		
**Diabetes**				
No	34.6	41.6	-7.0	0.001
Yes	29.2	30.9	-1.6	0.001
**Hypertension**				
No	34.1	40.4	-6.3	0.001
Yes	33.0	39.4	-6.4	0.001
**Stroke**				
No	33.6	40.0	-6.4	0.000
Yes	39.7	41.5	-1.8	0.307
**Heart Disease**				
No	33.8	40.5	-6.7	0.001
Yes	33.3	30.9	2.4	0.001
**Age group (in years)**				
Young-old (60–69)	28.9	36.5	-7.7	0.001
Old-old (70–79)	38.4	44.7	-6.3	0.001
Oldest-old (80 +)	47.8	47.4	0.4	0.290
**Education**				
No education	41.4	43.1	-1.8	0.001
Primary	34.7	36.8	-2.1	0.001
Secondary	26.2	23.5	2.7	0.758
Higher	18.9	26.2	-7.3	0.103
**Marital status**				
Currently married	32.3	38.0	-5.7	0.001
Widowed	38.6	41.8	-3.2	0.001
Others^a^	48.9	38.2	10.7	0.408
**Working status**				
Never worked	44.7	41.4	3.3	0.556
Currently working	29.9	38.0	-8.1	0.001
Not currently working	36.4	39.3	-3.0	0.001
**Currently smoke tobacco**				
No	33.9	40.0	-6.2	0.001
Yes	33.5	39.6	-6.1	0.001
**Currently chew tobacco**				
No	33.2	40.0	-6.8	0.001
Yes	35.4	40.2	-4.8	0.001
**Alcohol consumption**				
No	33.4	39.9	-6.5	0.001
Yes	34.8	44.5	-9.7	0.001
**MPCE quintile**				
Poorest	38.2	42.0	-3.9	0.001
Poorer	36.5	42.9	-6.5	0.001
Middle	32.8	40.1	-7.4	0.001
Richer	31.2	36.4	-5.3	0.001
Richest	29.2	37.5	-8.3	0.001
**Religion**				
Hindu	33.6	40.1	-6.6	0.001
Muslim	31.6	38.4	-6.8	0.001
Christian	40.9	42.6	-1.7	0.001
Others^b^	40.4	40.8	-0.5	0.136
**Caste**				
Scheduled Caste	40.0	43.2	-3.2	0.001
Scheduled Tribe	37.4	43.1	-5.6	0.001
Other Backward Class	33.4	38.9	-5.6	0.001
Others	29.0	37.9	-8.9	0.001
**Place of residence**				
Rural	36.3	42.6	-6.3	0.001
Urban	26.8	34.2	-7.4	0.001
**Region**				
North	35.0	47.6	-12.6	0.001
Central	34.4	39.3	-4.8	0.001
East	37.2	43.6	-6.4	0.001
Northeast	44.3	53.9	-9.6	0.001
West	23.6	30.0	-6.3	0.001
South	34.2	38.6	-4.3	0.001
**Overall**	**33.8**	**40.0**	**-6.2**	**0.001**

^a^ Includes Divorced/Separated/Deserted/Others

^b^ Includes Sikh, Buddhist/neo-Buddhist, Jain, Jewish, and Parsi/Zoroastrian; Differences: Male–Female

**Fig. 1:**
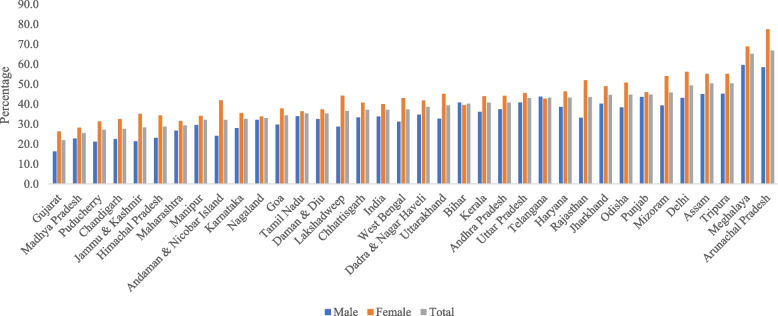
Prevalence of VI among older adults in India and its states, 2017–18

State-wise prevalence (%) of VI among older adults is presented in Fig. 1. Older males in Meghalaya (59.5%) had the highest prevalence of VI, followed by older males in Arunachal Pradesh (58.4%) and Tripura (45.2%). Additionally, older females in Arunachal Pradesh (77.4%) had the highest prevalence for VI, followed by Meghalaya (68.8%) and Delhi (56.1%). The state-wise prevalence of VI according to the 20/63 cut-off is provided in Fig. 1.

Logistic regression estimates for VI among older adults is presented in Table 4. Diabetic patients significantly had 18% lower odds of having VI than that of non-diabetic individuals [AOR: 0.82; CI: 0.70, 0.95]. On the other hand, older who were suffering from hypertension and stroke had 12% [AOR: 1.12; CI: 1.01, 1.22] and 20% [AOR: 1.20; CI: 1.03, 1.53] higher likelihood of having VI than their counterparts.

**Table 4:** Multivariable logistic regression estimates for VI among older adults, India, LASI Wave 1, 2017–18

Background characteristics	AOR (CI)
**Diabetes**	
No	1.00
Yes	0.82* (0.70,0.95)
**Hypertension**	
No	1.00
Yes	1.12*(1.01,1.22)
**Stroke**	
No	1.00
Yes	1.20*(1.03,1.53)
**Heart Disease**	
No	1.00
Yes	0.90 (0.67,1.22)
**Sex**	
Male	1.00
Female	0.98 (0.86,1.11)
**Age group (in years)**	
Young-old (60–69)	1.00
Old-old (70–79)	1.39*** (1.24,1.54)
Oldest-old (80 +)	1.58*** (1.32,1.89)
**Education**	
No education	1.00
Primary	0.82*** (0.73,0.92)
Secondary	0.53*** (0.43,0.64)
Higher	0.42*** (0.34,0.52)
**Marital status**	
Currently married	1.00
Widowed	1.01 (0.91,1.13)
Others^a^	1.42* (1.08,1.87)
**Working status**	
Never worked	1.00
Currently working	0.77*** (0.67,0.88)
Not currently working	0.89 (0.78, 1.01)
**Currently smoke tobacco**	
No	1.00
Yes	0.83** (0.73,0.95)
**Currently chew tobacco**	
No	1.00
Yes	0.97 (0.87,1.08)
**Alcohol consumption**	
No	1.00
Yes	1.03 (0.91,1.16)
**MPCE quintile**	
Poorest	1.00
Poorer	1.04 (0.92,1.18)
Middle	0.93 (0.81,1.06)
Richer	0.87 (0.75,1.00)
Richest	0.89 (0.75,1.05)
**Religion**	
Hindu	1.00
Muslim	0.85* (0.73,0.98)
Christian	1.12 (0.90,1.39)
Others^b^	1.08 (0.89,1.33)
**Caste**	
Scheduled Caste	1.00
Scheduled Tribe	0.94 (0.79,1.12)
Other Backward Class	0.89 (0.79,1.00)
Others	0.89 (0.78,1.01)
**Place of residence**	
Rural	1.00
Urban	0.86* (0.76,0.98)
**Region**	
North	1.00
Central	0.81** (0.71,0.92)
East	0.92 (0.81,1.04)
Northeast	1.34*** (1.14,1.57)
West	0.55*** (0.48,0.64)
South	0.86* (0.74,1.00)

^a^ Includes Divorced/Separated/Deserted/Others

^b^ Includes Sikh, Buddhist/neo-Buddhist, Jain, Jewish, and Parsi/Zoroastrian

*p* < 0.05*, *p* < 0.01**, *p* < 0.001***

Oldest old adults were 58% significantly more likely to suffer from VI than younger old adults [AOR: 1.58; CI: 1.32, 1.89]. Older adults with higher educational status had 58% significantly lower likelihood to suffer from VI than older adults with no education [AOR: 0.42; CI: 0.34, 0.52]. Separated/Divorced/Deserted/Others older adults had 42% significantly higher likelihood to suffer from VI than older adults who were currently married [AOR: 1.42; CI: 1.08, 1.87]. According to working status, currently working individuals had significantly lower odds of suffering from the VI than those who never worked [AOR: 0.77; CI: 0.67, 0.88]. It was found that higher the wealth quintile lowers the likelihood to suffer from lower vision problem among older adults. Older adults from urban place of residence had 14% significantly lower likelihood to suffer from VI than older adults from rural place of residence [AOR: 0.86; CI: 0.76, 0.98]. Older adults from northeast region had 34% significantly higher likelihood to suffer from VI than older adults from northern region [AOR: 1.34; CI: 1.14, 1.57]. The multivariable estimates according to the cut-point of worse than 20/63, are provided in Table S3.

## Discussion

In this study, based on a large representative survey data, we examined the association of chronic diseases, socioeconomic factors and health behaviours with VI among older adults. The substantially higher prevalence of VI (37 percent) among older adults aged 60 and above, that in most cases can be avoided with early detection and timely intervention, calls for special attention from the health decision makers in the country. The prevalence rate with a female disadvantage is comparable with other studies in India [26–29], and worldwide [4]. Moreover, the prevalence of blindness was 2.9% among Indian older adults which was lower than the prevalence (3.6%) reported in an earlier population-based survey of rapid assessment of avoidable blindness (RAAB) [30], and the pooled prevalence (4.17% for men and 5.68% for women) reported in a previous systematic review in India [31]. The variation in the prevalence of blindness in our study resulted from different method of assessment (we considered visual acuity of less than 3/60 in the better eye and being unable to count fingers or perceive light whereas, the systematic review also considered visual acuity < 6/60 in better eye, in the definition of blindness) and varied population age-group and time period. This variation can also be attributed to other socio-cultural and environmental and genetic factors [32] that are not considered in the current study.

Four major diseases related to VI were analysed and the prevalence of hypertension, and stroke were significantly positively associated with VI in our study. Hypertensive patients having higher odds of VI may be attributed to their improper compliance with medicines that leads to ophthalmic complications [33]. Hypertensive retinopathy is also a known complication that may also explain the current finding [34, 35]. Hypertension is reported as the most prevalent risk factor for stroke [36] which can also contribute to the increased risk of VI. Several previous studies have also examined VI as an index condition in conjunction with many of the chronic conditions [37–39]. Our results were parallel to the findings of earlier studies showing post-stroke induced VI in older ages [40–42]. Multiple studies have reported that older people with lowered vision and sensory impairments had increased risk for cardiovascular diseases and such diseases lie on the pathway from VI to mortality [43–45]. Also, with regard to the relationship between vision-related problems and health outcomes, study found that 74.5 percent of older people with VI had co-occurrence of at least one of the chronic conditions [46, 47]. Similarly, higher prevalence of VI and blindness in multi-morbidity studies was highlighted in a systematic review of 41 geriatric studies [48], suggesting the possibility of reverse causality among the observed associations.

On the contrary, the prevalence of diabetes among older individuals was negatively associated with VI in our study which is at variance with previous studies showing a positive association of diabetes mellitus with VI and blindness [49, 50]. This unexpected finding may be explained by the fact that older adults who self-reported diagnosis of diabetes might have received routine medical care and eye exams and utilized vision enhancing interventions like eyeglasses and cataract surgery as well as were more likely to have proper compliance with medicine [51]. However, since diabetes is known to be an important cause of VI and blindness in India and across the globe [52], the current finding requires further investigation.

Furthermore, association of marital status as a social support typically found in epidemiological studies with VI suggests an increased risk for VI among elders who are divorced/ separated/ deserted or never married, which is also observed in previous studies [53]. For them, a scarcity of assistance may result from experiencing loss of vision that calls for a special attention to be paid. The results of the present study are also concomitant with previous studies on the association of increasing age with higher rate of vision-related problems and blindness [54]. Besides, consistent with a few studies in different parts of India, VI was highly prevalent among low socioeconomic strata and in poorest wealth quintile [29, 55]. The significant negative association of VI with educational status was also observed in earlier studies [56]. Information regarding accessibility and barriers to eye care services which is lacking in the current study might have helped to better understand the relatively higher odds of VI among the illiterate older adults.

Higher rates of VI among rural resident older people aged 60 years and above in our study was consistent with multiple studies in various parts of the country showing an urban–rural gradient of poor vision in older ages and higher prevalence in rural areas [30, 57, 58]. The significant rural–urban difference observed in this study can be attributed to more limited access to eye care services including cataract surgery in rural areas as compared to urban areas, which has been documented extensively in the literature [59–61]. A community-based study in South India found that visual and hearing impairment are important health problems among older population in this region [62], suggesting a need for future studies with special focus on regional disparities in geriatric impairments including VI. Further, when looking at geographical differences, the increased prevalence of VI in northern and north-eastern regions are in variance with earlier studies in India showing more prevalence in southern and western regions of the country [27, 57]. Further studies with multi-level spatial analyses are warranted to explore the variations in visual impairment across regions and socioeconomic strata of older population in India.

Although the strengths of this study lie in its nationally representative sample of older adults aged 60 years and above and the use of measured prevalence of VI, several limitations are important to be acknowledged. All data regarding chronic conditions were based on respondents’ self- reports. Besides, causality cannot be established given the cross- sectional design. Moreover, reasons for vision-related problems should be explored separately for types of eye diseases using longitudinal and appropriate clinical studies.

## Conclusion

The present analysis identified higher rates of VI among those who are diagnosed with hypertension or stroke, currently unmarried, socioeconomically poorer, less educated and urban resident older people that can inform strategies to engage high risk groups. The findings outline possibilities to update information that can be utilized in development and improvement of vision in an aging population. Also, it suggests that specific interventions that promote active aging are required for older individuals who are socioeconomically disadvantaged as well as visually impaired.

## Supplementary Information

**Additional file 1: Table S1. **Age-sex adjusted and unadjusted prevalence of low vision among older adults in India, 2017–2018.** Table S2. **Prevalence (%) of VI among older adults according to background characteristics by sex, India, LASI Wave 1, 2017-18.** Table S3.** Multivariable logistic regression estimates for VI among older adults, India, LASI Wave 1, 2017-18.

## Data Availability

The datasets generated and/or analysed during the current study are available in the Gateway to Global Aging Data, https://g2aging.org/.

## Abbreviations

VI: Vision impairment
AOR: Adjusted Odds Ratio
CI: Confidence Interval
MPCE: Monthly per capita expenditure
VIF: Variance inflation factor
