# Supplementary material for: Prevalence and predictors of vision impairment among older adults in India: evidence from LASI, 2017–18
Source: BMC Ophthalmol. 2023 Jun 5;23:251. doi: 10.1186/s12886-023-03009-w (PMC10240694; doi:10.1186/s12886-023-03009-w)
Supplement: Supplementary file 1 — Additional file 1: Table S1. Age-sex adjusted and unadjusted prevalence of low vision among older adults in India, 2017–2018. Table S2. Prevalence (%) of VI among older adults according to background characteristics by sex, India, LASI Wave 1, 2017-18. Table S3. Multivariable logistic regression estimates for VI among older adults, India, LASI Wave 1, 2017-18. [file 12886_2023_3009_MOESM1_ESM.docx]

| **Table S1:** Age-sex adjusted and unadjusted prevalence of low vision among older adults in India, 2017–2018 | | | | |
| --- | --- | --- | --- | --- |
|  | **Unadjusted** | | **Adjusted** | |
|  | **%** | **CI** | **%** | **CI** |
| **Overall** | **64.7** | **(64.2, 65.3)** | **64.1** | **(63.6, 64.7)** |
|  |  |  |  |  |
| **Diabetes** |  |  |  |  |
| No | 65.5 | (64.9, 66.1) | 65.4 | (64.8, 66) |
| Yes | 60.6 | (59.2, 62.1) | 56.4 | (54.9, 57.9) |
| **Hypertension** |  |  |  |  |
| No | 65.5 | (64.8, 66.2) | 64.6 | (63.9, 65.3) |
| Yes | 63.3 | (62.3, 64.2) | 63.1 | (62.2, 64.1) |
| **Stroke** |  |  |  |  |
| No | 64.7 | (64.1, 65.2) | 64.0 | (63.5, 64.6) |
| Yes | 67.0 | (63.4, 70.7) | 66.7 | (63.2, 70.2) |
| **Heart Disease** |  |  |  |  |
| No | 65.0 | (64.4, 65.5) | 64.4 | (63.8, 65) |
| Yes | 60.8 | (58.3, 63.4) | 59.1 | (56.6, 61.7) |
| **Education** |  |  |  |  |
| No education | 69.3 | (68.6, 70.1) | 69.0 | (68.2, 69.7) |
| Primary | 64.6 | (63.5, 65.8) | 63.8 | (62.7, 65) |
| Secondary | 57.1 | (55.3, 58.9) | 52.8 | (51.3, 54.4) |
| Higher | 49.7 | (46.9, 52.4) | 47.7 | (45.5, 49.8) |
| **Marital status** |  |  |  |  |
| Currently married | 64.2 | (63.5, 65) | 63.2 | (62.5, 63.9) |
| Widowed | 66.6 | (65.3, 67.9) | 65.4 | (64.4, 66.4) |
| Others^1^ | 65.7 | (62.3, 69.2) | 68.8 | (65.4, 72.2) |
| **Working status** |  |  |  |  |
| Never worked | 70.0 | (68.1, 71.8) | 66.1 | (65, 67.1) |
| Currently working | 65.3 | (64, 66.6) | 61.9 | (60.9, 63) |
| Not currently working | 63.4 | (62.5, 64.3) | 64.5 | (63.7, 65.4) |
| **Smoke tobacco** |  |  |  |  |
| No | 64.2 | (63.6, 64.8) | 64.3 | (63.7, 64.9) |
| Yes | 67.7 | (65.8, 69.6) | 63.1 | (61.6, 64.5) |
| **Chew tobacco** |  |  |  |  |
| No | 64.3 | (63.7, 64.9) | 63.9 | (63.3, 64.6) |
| Yes | 66.6 | (65.4, 67.9) | 64.8 | (63.5, 66) |
| **Alcohol consumption** |  |  |  |  |
| No | 64.3 | (63.7, 64.9) | 64.2 | (63.5, 64.8) |
| Yes | 66.9 | (64.9, 68.9) | 63.8 | (62.5, 65.1) |
| **MPCE quintile** |  |  |  |  |
| Poorest | 66.8 | (65.6, 68) | 66.5 | (65.3, 67.7) |
| Poorer | 67.2 | (66, 68.4) | 67.6 | (66.4, 68.8) |
| Middle | 64.8 | (63.6, 66.1) | 64.6 | (63.4, 65.8) |
| Richer | 63.4 | (62.1, 64.6) | 60.8 | (59.6, 62.1) |
| Richest | 61.3 | (60, 62.6) | 59.8 | (58.5, 61.1) |
| **Religion** |  |  |  |  |
| Hindu | 64.3 | (63.6, 64.9) | 64.0 | (63.3, 64.6) |
| Muslim | 62.3 | (60.7, 64) | 62.9 | (61.3, 64.5) |
| Christian | 68.1 | (66.3, 69.8) | 67.8 | (66.1, 69.5) |
| Others^2^ | 70.6 | (68.1, 73) | 68.7 | (66.3, 71.2) |
| **Caste** |  |  |  |  |
| Scheduled Caste | 68.5 | (67.1, 69.8) | 68.7 | (67.4, 70.1) |
| Scheduled Tribe | 68.0 | (66.7, 69.3) | 67.7 | (66.4, 69.1) |
| Other Backward Class | 63.2 | (62.3, 64.1) | 62.0 | (61.1, 63) |
| Others | 62.3 | (61.2, 63.4) | 62.7 | (61.6, 63.8) |
| **Place of residence** |  |  |  |  |
| Rural | 67.2 | (66.6, 67.9) | 66.6 | (65.9, 67.2) |
| Urban | 59.8 | (58.8, 60.7) | 58.0 | (57, 59) |
| **Region** |  |  |  |  |
| North | 68.0 | (66.8, 69.3) | 69.9 | (68.7, 71.2) |
| Central | 61.7 | (60.2, 63.3) | 63.7 | (62.2, 65.3) |
| East | 68.6 | (67.4, 69.8) | 68.8 | (67.6, 70.1) |
| Northeast | 73.4 | (71.9, 74.9) | 75.5 | (74.1, 77) |
| West | 55.1 | (53.5, 56.7) | 53.9 | (52.3, 55.4) |
| South | 61.9 | (60.7, 63) | 62.1 | (60.9, 63.3) |
| *Note:* 1 – includes Divorced/Separated/Deserted/Others; 2 – includes Sikh, Buddhist/neo-Buddhist, Jain, Jewish, and Parsi/Zoroastrian | | | | |

| **Table S2.** Prevalence (%) of VI among older adults according to background characteristics by sex, India, LASI Wave 1, 2017-18 | | | | |
| --- | --- | --- | --- | --- |
| **Background characteristics** | **Male** | **Female** | **Difference** | **p-value** |
|  | **%** | **%** |  |  |
| **Diabetes** |  |  |  |  |
| No | 62.8 | 67.8 | -5.0 | <0.001 |
| Yes | 58.2 | 54.7 | 3.5 | <0.001 |
| **Hypertension** |  |  |  |  |
| No | 62.6 | 66.7 | -4.1 | <0.001 |
| Yes | 60.9 | 64.6 | -3.7 | <0.001 |
| **Stroke** |  |  |  |  |
| No | 62.0 | 65.9 | -3.9 | <0.001 |
| Yes | 67.5 | 65.8 | 1.7 | 0.910 |
| **Heart Disease** |  |  |  |  |
| No | 62.0 | 66.5 | -4.5 | <0.001 |
| Yes | 64.2 | 53.5 | 10.7 | <0.001 |
| **Age group (in years)** |  |  |  |  |
| Young-old (60-69) | 58.5 | 64.4 | -5.9 | <0.001 |
| Old-old (70-79) | 65.9 | 67.7 | -1.8 | <0.001 |
| Oldest-old (80+) | 71.5 | 69.3 | 2.2 | 0.791 |
| **Education** |  |  |  |  |
| No education | 68.2 | 69.3 | -1.1 | 0.030 |
| Primary | 63.8 | 63.9 | -0.1 | 0.015 |
| Secondary | 56.1 | 45.6 | 10.5 | 0.769 |
| Higher | 47.8 | 47.0 | 0.8 | 0.389 |
| **Marital status** |  |  |  |  |
| Currently married | 61.5 | 65.9 | -4.4 | <0.001 |
| Widowed | 63.9 | 65.8 | -1.9 | 0.002 |
| Others^1^ | 70.2 | 67.2 | 3.0 | 0.609 |
| **Working status** |  |  |  |  |
| Never worked | 71.1 | 65.7 | 5.4 | 0.056 |
| Currently working | 60.2 | 65.6 | -5.4 | <0.001 |
| Not currently working | 63.2 | 66.4 | -3.2 | <0.001 |
| **Currently smoke tobacco** |  |  |  |  |
| No | 62.0 | 65.9 | -3.9 | <0.001 |
| Yes | 62.5 | 66.8 | -4.3 | <0.001 |
| **Currently chew tobacco** |  |  |  |  |
| No | 61.7 | 65.7 | -4.0 | <0.001 |
| Yes | 63.4 | 67.0 | -3.6 | <0.001 |
| **Alcohol consumption** |  |  |  |  |
| No | 61.7 | 65.8 | -4.1 | <0.001 |
| Yes | 63.3 | 69.1 | -5.8 | <0.001 |
| **MPCE quintile** |  |  |  |  |
| Poorest | 64.4 | 68.2 | -3.8 | 0.001 |
| Poorer | 65.5 | 69.4 | -3.9 | <0.001 |
| Middle | 62.4 | 66.6 | -4.2 | <0.001 |
| Richer | 59.4 | 62.1 | -2.7 | <0.001 |
| Richest | 57.9 | 61.6 | -3.7 | <0.001 |
| **Religion** |  |  |  |  |
| Hindu | 62.2 | 65.6 | -3.4 | <0.001 |
| Muslim | 58.4 | 67.1 | -8.7 | <0.001 |
| Christian | 67.4 | 68.0 | -0.6 | 0.000 |
| Others^2^ | 68.9 | 68.6 | 0.3 | 0.622 |
| **Caste** |  |  |  |  |
| Scheduled Caste | 66.5 | 70.8 | -4.3 | <0.001 |
| Scheduled Tribe | 66.2 | 68.9 | -2.7 | <0.001 |
| Other Backward Class | 61.0 | 63.0 | -2.0 | <0.001 |
| Others | 60.1 | 65.1 | -5.0 | <0.001 |
| **Place of residence** |  |  |  |  |
| Rural | 64.9 | 68.1 | -3.2 | <0.001 |
| Urban | 54.5 | 60.7 | -6.2 | <0.001 |
| **Region** |  |  |  |  |
| North | 64.5 | 74.6 | -10.1 | <0.001 |
| Central | 62.0 | 65.6 | -3.6 | 0.154 |
| East | 66.7 | 70.9 | -4.2 | <0.001 |
| Northeast | 71.7 | 79.0 | -7.3 | <0.001 |
| West | 50.9 | 56.2 | -5.3 | <0.001 |
| South | 62.4 | 61.9 | 0.5 | <0.001 |
| **Overall** | **62.1** | **65.9** | **-3.8** | <0.001 |
| *Note*: 1 – includes Divorced/Separated/Deserted/Others; 2 – includes Sikh, Buddhist/neo-Buddhist, Jain, Jewish, and Parsi/Zoroastrian; Differences: Male-Female | | | | |

| **Table S3.** Multivariable logistic regression estimates for VI among older adults, India, LASI Wave 1, 2017-18 | |
| --- | --- |
| **Background characteristics** | **AOR (CI)** |
| **Diabetes** |  |
| No | 1 |
| Yes | 0.82* (0.70,0.97) |
| **Hypertension** |  |
| No | 1 |
| Yes | 1.07 (0.97,1.18) |
| **Stroke** |  |
| No | 1 |
| Yes | 1.18 (0.91,1.52) |
| **Heart Disease** |  |
| No | 1 |
| Yes | 0.94 (0.67,1.33) |
| **Sex** |  |
| Male | 1 |
| Female | 0.97 (0.85,1.09) |
| **Age group (in years)** |  |
| Young-old (60-69) | 1 |
| Old-old (70-79) | 1.22*** (1.08,1.37) |
| Oldest-old (80+) | 1.33** (1.11,1.59) |
| **Education** |  |
| No education | 1 |
| Primary | 0.86** (0.77,0.96) |
| Secondary | 0.54*** (0.44,0.66) |
| Higher | 0.44*** (0.36,0.54) |
| **Marital status** |  |
| Currently married | 1 |
| Widowed | 0.91 (0.79,1.05) |
| Others^1^ | 1.01 (0.87,1.16) |
| **Working status** |  |
| Never worked | 1 |
| Currently working | 0.89 (0.79,1.01) |
| Not currently working | 1.24 (0.95,1.61) |
| **Currently smoke tobacco** |  |
| No | 1 |
| Yes | 0.84** (0.74,0.96) |
| **Currently chew tobacco** |  |
| No | 1 |
| Yes | 0.94 (0.84,1.04) |
| **Alcohol consumption** |  |
| No | 1 |
| Yes | 1.02 (0.90,1.15) |
| **MPCE quintile** |  |
| Poorest | 1 |
| Poorer | 1.1 (0.97,1.25) |
| Middle | 0.99 (0.87,1.12) |
| Richer | 0.89 (0.76,1.03) |
| Richest | 0.87 (0.74,1.03) |
| **Religion** |  |
| Hindu | 1 |
| Muslim | 0.87* (0.75,1.00) |
| Christian | 1.05 (0.83,1.33) |
| Others^2^ | 1.15 (0.94,1.41) |
| **Caste** |  |
| Scheduled Caste | 1 |
| Scheduled Tribe | 0.93 (0.78,1.11) |
| Other Backward Class | 0.85** (0.75,0.95) |
| Others | 0.98 (0.85,1.12) |
| **Place of residence** |  |
| Rural | 1 |
| Urban | 0.9 (0.81,1.01) |
| **Region** |  |
| North | 1 |
| Central | 0.77*** (0.67,0.88) |
| East | 0.93 (0.82,1.05) |
| Northeast | 1.38*** (1.16,1.63) |
| West | 0.53*** (0.46,0.61) |
| South | 0.79** (0.68,0.92) |
| *Note*: 1 – includes Divorced/Separated/Deserted/Others; 2 – includes Sikh, Buddhist/neo-Buddhist, Jain, Jewish, and Parsi/Zoroastrian; p<0.05*, p<0.01**,p<0.001*** | |
